# Crosstalk between ERα and Receptor Tyrosine Kinase Signalling and Implications for the Development of Anti-Endocrine Resistance

**DOI:** 10.3390/cancers10060209

**Published:** 2018-06-20

**Authors:** Rugaia Z. Montaser, Helen M. Coley

**Affiliations:** Faculty of Health and Medical Sciences, University of Surrey, Guildford, Surrey GU2 7XH, UK; rugaia.montaser@gmail.com

**Keywords:** breast cancer, drug resistance, receptor tyrosine kinases

## Abstract

Although anti-endocrine therapies have significantly advanced the treatment of breast cancer, they pose the problem of acquired drug resistance. The oestrogen receptor (ER)-expressing breast cancer cell lines MCF-7 and T47D alongside their in vitro derived resistant counterparts MCF-7-TR (tamoxifen-resistant) and T47D-FR (fulvestrant-resistant) showed dual resistance to fulvestrant and tamoxifen in the presence of upregulated HER1 and HER2 growth factor receptors. Our study demonstrated that tamoxifen resistance and fulvestrant resistance are associated with collateral sensitivity to the tyrosine kinase inhibitors (TKIs) lapatinib (*p* < 0.0001) and afatinib (*p* < 0.0001). Further, we found that over time, the TKIs reactivated ERα protein and/or mRNA in tamoxifen- and fulvestrant-resistant cells. Combinations of anti-endocrine agents with afatinib gave rise to significantly enhanced levels of apoptosis in both T47D-FR and MCF-7-TR in a synergistic manner versus additive effects of agents used singly. This was associated with p27^kip1^ induction for anti-endocrine-resistant cells versus parental cells. Our data supports the use of combination treatment utilising dual HER1/2 inhibitors in breast cancer patients showing resistance to multiple anti-endocrine agents.

## 1. Introduction 

The oestrogen receptor-α (ERα) signalling pathways and their associated ligands are key players in breast cancer progression. ERα is a nuclear ligand-dependent transcriptional factor that can communicate most mitogenic and survival stimuli in cancerous breast tissue through genomic and non-genomic actions [1]. This activity is mediated by the interaction between a small fraction of ER and various components of growth factor receptor tyrosine kinase signalling pathways [1,2]. Growth factor receptor signalling pathways are frequently altered in cancer and are a significant component of the hallmarks of the cancer model as proposed by Hanahan and Weinberg [3,4]. Cumulative clinical data suggest aberrant HER1 (EGFR) and HER2 signalling is causally associated with increased cancer cell progression, shorter survival, and a poor outcome for patients with breast carcinomas [5]. Further, the membrane tyrosine kinase *HER2* gene is amplified in 20–25% of ER-positive breast cancers [6].

Tamoxifen resistance poses a significant problem in the management of ER-positive breast cancers, with one-third of women treated with this agent for 5 years relapsing with recurrent disease within 15 years [7]. Anti-endocrine resistance can occur when oestrogen and tamoxifen activate cytoplasmic ERα, which can then result in phosphorylation and activation of surface tyrosine kinase receptors of the HER family [8,9]. These interactions lead to the activation of key downstream signalling kinases such as PI3K, MAPK, and AKT, which also have the potential to phosphorylate and thereby activate ERα itself or its co-activator proteins [10,11], further resulting in enhanced expression of oestrogen-regulated genes [12].

Detailed knowledge of these processes is incomplete, but factors such as expression of the HER1 and HER2 receptors, alterations in upstream regulators along with decreased expression of ERα, and up-regulation of key cell-cycle regulators (e.g., MYC and D- and E-cyclins) lead to the induction of mitogenic signalling pathways and suppression of apoptosis [13,14].

In order to address these issues further, we studied two ER-positive breast cancer cell lines alongside their tamoxifen- and fulvestrant-resistant variants. To ascertain any therapeutic opportunities for the improved management of drug-resistant breast cancer, we looked at the approach of using combinations of anti-endocrine agents with a variety of tyrosine kinase inhibitors (TKIs) directed against HER1 and HER2.

## 2. Results

### 2.1. Levels of Anti-Endocrine Resistance in Breast Cancer Cell Lines

**Figure 1:**
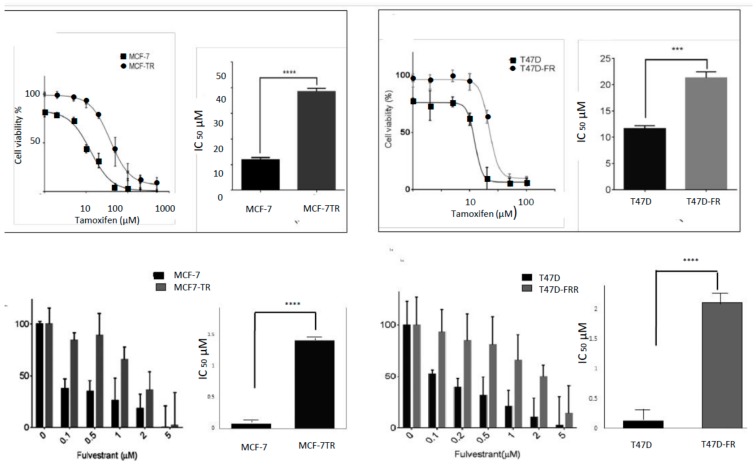
Assessment of tamoxifen (**upper panels**) and fulvestrant (**lower panels**) sensitivity in breast cancer cell lines. An MTT assay was used to measure sensitivity to tamoxifen and a clonogenic assay was used for assessment of fulvestrant sensitivity (*n* ≥ 4). Dose–response curves were fitted using the Prism (version 6) program. IC_50_ values indicating the levels of drug resistance are shown by bar graphs using a paired *t*-test: *** *p* < 0.001; **** *p* < 0.0001.

An assessment of the sensitivities of the cell lines to both tamoxifen and fulvestrant is shown in Figure 1, with fold resistance calculated as the IC_50_ resistant line/IC_50_ parental line. For technical reasons, it was not possible to use an MTT assay to measure the sensitivity of the breast cancer cell lines to fulvestrant, as false levels of cell proliferation were indicated, in line with the findings of others [15]. For this reason, we carried out a clonogenic assay for the assessment of fulvestrant sensitivity. Whilst MCF7-TR showed approximately 3-fold resistance to tamoxifen (IC_50_ values of 11.91 ± 1.86 µM and 35.5 ± 1.26 µM for MCF-7 and MCF7-TR, respectively), it also showed approximately 17-fold cross-resistance to fulvestrant (IC_50_ values of 0.08 ± 0.02 µM and 1.41 ± 0.24 µM for MCF-7 and MCF-7-TR, respectively); likewise the T47D-FR cell line showed approximately 16-fold resistance to fulvestrant (IC_50_ values of 0.13 ± 0.04 µM and 2.10 ± 0.34 µM for T47D and T47D-FR, respectively) and approximately 2-fold cross-resistance to tamoxifen (IC_50_ values of 11.0 ± 0.70 µM and 21.40 ± 0.84 µM for T47D and T47D-FR, respectively).

### 2.2. Development of Anti-Endocrine-Resistant Breast Cancer Cells Gives Rise to Changes in Expression of HER Receptors

**Figure 2:**
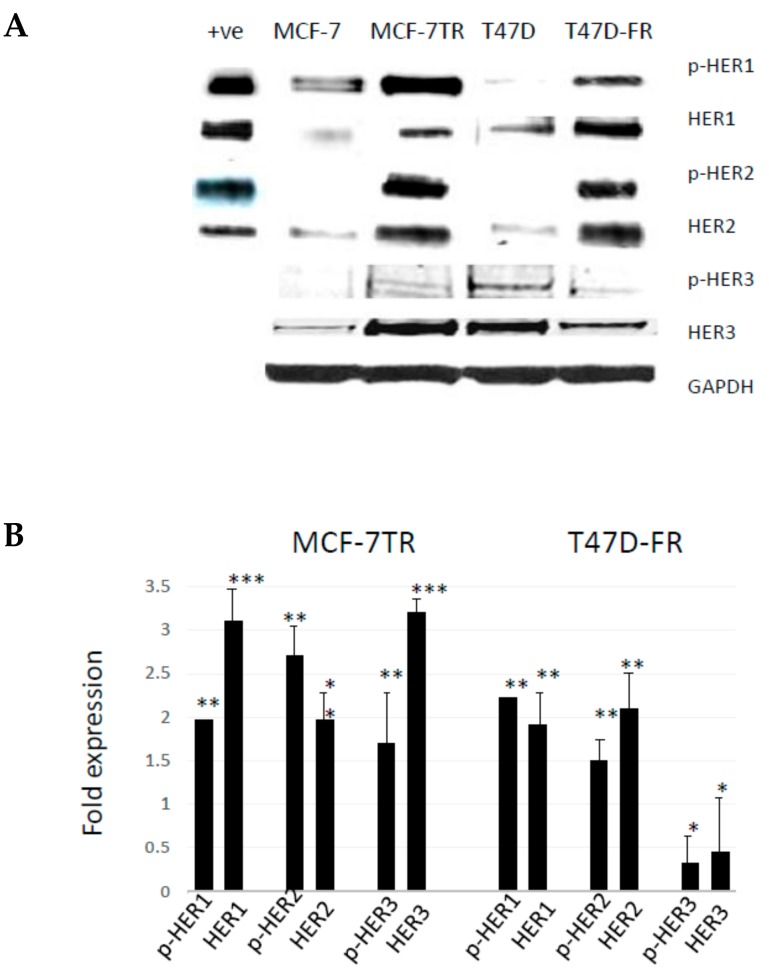
Levels of HER expression in breast cancer cell lines with acquired resistance to anti-oestrogen therapy. Representative Western blot data obtained from whole-cell lysates (**A**) separated by SDS-PAGE and immunoblotted onto PVDF membranes before immunodetection (*n* = 4). The head and neck cancer HN5 cell line HER1 + ve and breast cancer SKBR3 cell line HER2 + ve were used as positive controls. (**B**) Expression levels of parental cells (set at 1.0) were compared with the resistant variant using the following paired *t*-test levels of statistical significance for densitometric scans: * *p* < 0.05; ** *p* < 0.001; *** *p* < 0.0001.

Figure 2 shows the relative protein expression levels of HER receptors in parental and anti-endocrine-resistant MCF-7 and T47D cell lines using Western blotting. HER1 was detected at lower levels in the parental MCF-7 and T47D cells, whilst there were relatively higher levels of activated pHER1 for both MCF-7 lines versus T47D lines. However, pHER1 levels were significantly higher in resistant versus parental lines in the resistant MCF-7-TR and T47D-FR cells. HER2 was found to be upregulated in both resistant cell lines (MCF-7-TR and T47D-FR). We measured the expression levels of HER3 to assess if more was available to form a possible heterodimer with HER2. Figure 2 shows higher expression in MCF-7-TR cells compared with MCF-7 cells, but the level was somewhat lower in the fulvestrant-resistant T47D-FR line compared with parental T47D cells. Expression of p-HER3 was similar to that of the native form, with relatively higher levels in MCF-7-TR versus MCF-7 and relatively lower levels in T47D-FR versus T47D.

### 2.3. Anti-Endocrine-Resistant Cells Show Collateral Sensitivity to TKI Therapies

**Figure 3:**
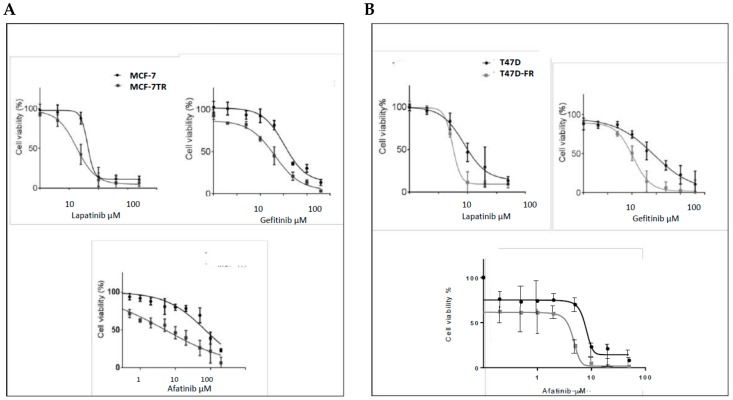
Sensitivity of breast cancer cell lines with acquired resistance to anti-oestrogen therapy to tyrosine kinase inhibitors (TKIs), assessed by MTT assay (*n* ≥ 4), (**A**) MCF-7 and (**B**) T47D cell lines. Dose–response curves were fitted using the Prism (version 6) program. Data show a broad collateral sensitivity of anti-endocrine-resistant cells to lapatinib, gefitinib, and afatinib. See Section 2.3 for statistical analyses.

Using the MTT assay, the responses of parental MCF-7 cells and tamoxifen-resistant MCF-7-TR cells to lapatinib, gefitinib, and afatinib were determined, as shown in Figure 3A,B. The dose–response curves obtained confirmed that MCF-7-TR cells showed collateral sensitivity to TKIs, as indicated by the following IC_50_ values (MCF-7 versus MCF-7-TR):

Lapatinib: 7.00 ± 0.25 µM versus 3.5 ± 0.50 µM; gefitinib: 18.25 ± 3.18 µM versus 34.20 ± 1.69 µM; afatinib: 7.09 ± 2.12 µM versus 0.50 ± 1.88 µM. The differences in responses to these drugs were highly statistically significant by the *t*-test (*p* < 0.05, *p* < 0.05, and *p* < 0.001, respectively).

As for MCF-7-TR, the T47D-FR cell line showed collateral sensitivity to TKIs (Figure 3B). Similar data obtained for parental T47D versus fulvestrant-resistant T47D-FR cells were as follows: lapatinib: 8.4 ± 0.84 µM versus 3.32 ± 1.3 µM; gefitinib: 29.0 ± 1.41 µM versus 8.40 ± 0.84 µM; afatinib: 5.4 ± 0.51 µM versus 0.95 ± 0.35 µM. All were statistically significant by the *t*-test (*p* < 0.01, *p* < 0.05, and *p* < 0.01, respectively). Hence, the dual TKIs versus gefitinb (HER1) showed the most significant effects in the drug-resistant cell lines.

### 2.4. HER1 and HER2 Expression in Endocrine-Resistant Cell Lines Following TKI Treatment (Confocal Microscopy)

**Figure 4:**
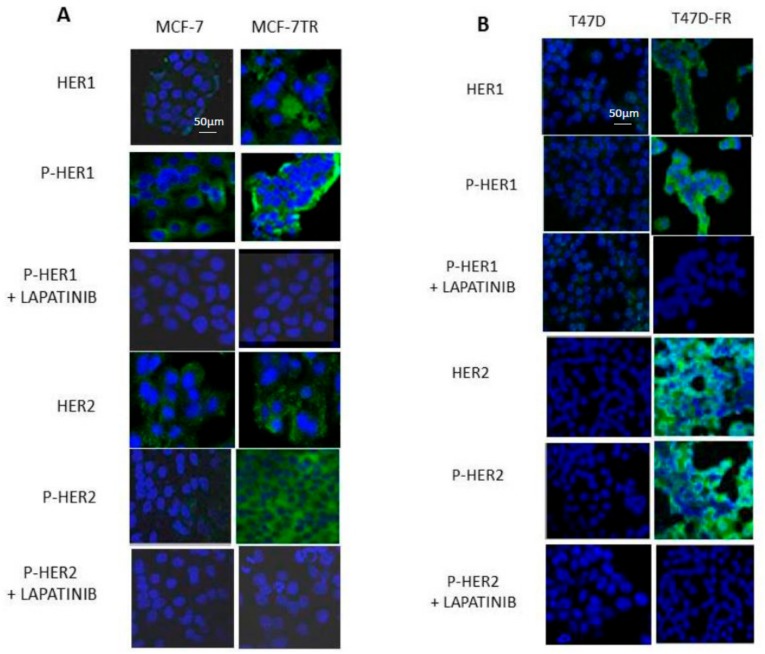
HER1 and HER2 expression with and without tyrosine kinase inhibitor (TKI) treatment in parental MCF-7 and MCF-7-TR (tamoxifen-resistant) and T47D and T47D-FR (fulvestrant-resistant) breast cancer cell lines using confocal fluorescence microscopy. Cells were treated with 5 µM lapatinib and incubated for 48 h. TO-PRO-3 (blue) was used to label nuclei, and HER1 (**A**) and HER2 (**B**) antibody reactions were conjugated to Alexa-Fluor 488 secondary antibody. Images are representative of at least three separate experiments. Settings for the microscope were maintained as consistently as possible from one experiment to another.

Figure 4A shows that HER1 and pHER1 levels in the parental MCF-7 cells were lower than for MCF-7-TR before treatment, with both diffuse cytoplasmic and membranous locations. Lapatinib treatment (5 µM) reduced the levels of pHER1 to similar levels as for both MCF-7 and MCF-7-TR. In MCF-7-TR, there were higher amounts of native and phosphorylated active forms of HER2 compared with MCF-7, in line with the findings shown in Figure 2. Upon treatment with lapatinib, pHER2 levels decreased in MCF-7-TR cells to the extent to which no associated fluorescence was seen and images for parent and resistant cells were closely matched.

Figure 4B shows the expression levels of HER1 and HER2 in parental and endocrine-resistant T47D cells using confocal microscopy, in line with the Western blot data in Figure 2. Images indicate markedly upregulated levels of total and membranous pHER1 and pHER2 in the resistant T47D-FR cells compared with parental cells, which diminished upon treatment with 5 µM lapatinib.

### 2.5. TKI Therapy Amplifies p27^kip1^ in the Presence of Anti-Endocrine Therapies as a Mechanism of Overcoming Drug Resistance

**Figure 5:**
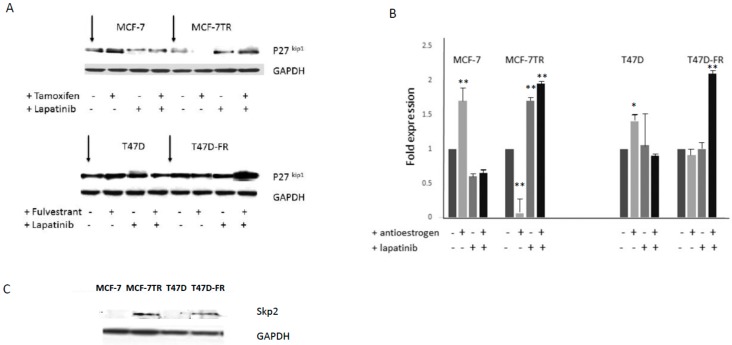
(**A**) Levels of p27^kip1^ expression in drug-sensitive and -resistant MCF-7 and T47D breast cancer cell lines in the absence or presence of the 5 µM lapatinib and anti-oestrogen therapy, with densitometry levels shown (**B**). (**C**) Constitutive expression of Skp2 in breast cancer cell lines. Representative Western blot data obtained from whole-cell lysates separated by SDS-PAGE, immunoblotted onto PVDF membranes before immunodetection (*n* = 3). Expression levels were set as 1.0 (control cells) using densitometric analysis and are then shown in response to the various drug treatments relative to each control: * *p* < 0.05; ** *p* < 0.001.

Following treatment of the cell lines in the absence or presence of drugs, administered singly or in combination, Western blot data was attained (Figure 5A,B). Tamoxifen or fulvestrant treatment alone clearly gave rise to p27^kip1^ induction for parental lines, but this response was absent from both of the drug-resistant variants. Treatment with lapatinib gave rise to p27^kip1^ induction for both drug-sensitive and -resistant variants. This was particularly increased in fulvestrant-resistant T47D-FR when treated with the drug combination. Figure 5C indicates the levels of the ubiquitination pathway protein Skp2, which clearly demonstrated high levels in tamoxifen- and fulvestrant-resistant cells, in line with the relatively lower levels of its target protein p27^kip1^.

### 2.6. The Impact of TKI Treatment on ERα Expression in Drug-Resistant Cells

**Figure 6:**
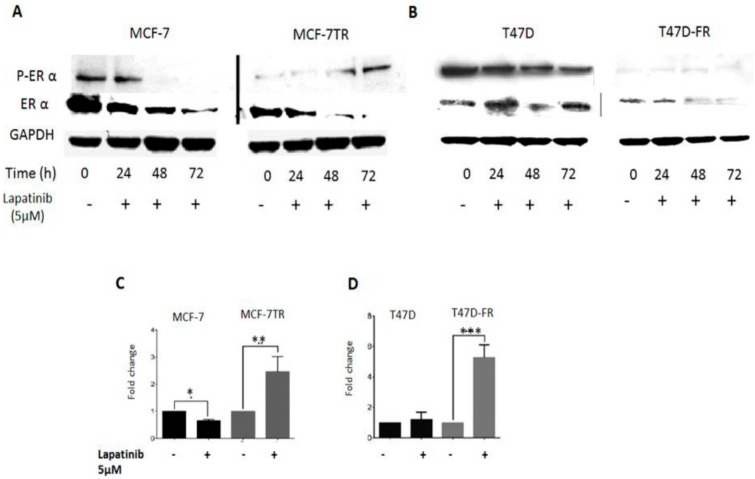
Oestrogen receptor-α (ERα) re-activation following treatment of anti-oestrogen-resistant breast cancer cell lines with lapatinib. (**A**,**B**) Representative Western blots of ERα and activated pERα at time points up to 72 h for MCF-7 and T47D cell lines, respectively. Representative Western blot data obtained from whole-cell lysates (upper panel) separated by SDS-PAGE, immunoblotted onto PVDF membranes before immunodetection (*n* = 3). (**C**,**D**) Levels of fold change in ESR mRNA as measured by qPCR and corrected to GAPDH as the housekeeping gene, using the ΔΔC_t_ equation. Expression in MCF-7 (**C**) and T47D (**D**) parental cell line control (untreated) values were normalised to 1.0. Statistical analysis was performed using a two-tailed *t*-test: * *p* < 0.05; ** *p* < 0.01.

In this set of experiments, we investigated the effect of TKI treatment on ERα status to assess whether there was evidence of crosstalk between growth factor receptors and ERα receptor in anti-endocrine-resistant MCF-7 and T47D cells (Figure 6). The control (untreated) lanes clearly showed the ER status of the parental lines (presence of native and activated forms of ERα) and anti-endocrine-resistant variants (markedly reduced levels of both forms of ERα). Following treatment with 5 µM lapatinib up to 72 h, Western blotting revealed that phosphorylated ERα expression was gradually reactivated in tamoxifen-resistant cells and not in parental cells. In the same experiments, fulvestrant-resistant T47D-FR cells showed consistently very low pERα expression, and expression gradually reduced over time in parental T47D cells.

Figure 6C,D shows qPCR analysis of ERα mRNA levels in MCF-7 and T47D parent and resistant cells after 72 h treatment with lapatinib (5 µM). Lapatinib caused a 2.5-fold increase in the mRNA level of ERα in the tamoxifen-resistant cells compared with the untreated control (*p* < 0.01); in contrast, the ERα mRNA level was reduced in the lapatinib-treated MCF-7 cells compared with the untreated control (*p* < 0.05) (Figure 6C). Corresponding ERα mRNA levels in T47D and T47D-FR cells showed a significant increase in fulvestrant-resistant cells treated with lapatinib compared with the untreated control (*p* < 0.001). At the protein level, little expression was seen; this could have been a reflection of the long doubling time of the T47D-FR line. Treatment with lapatinib had no significant effect in parental T47D cells compared to the untreated control.

### 2.7. The Co-Administration of TKI and Anti-Oestrogens Enhances Cell Death and Overcomes Anti-Endocrine Resistance

**Figure 7:**
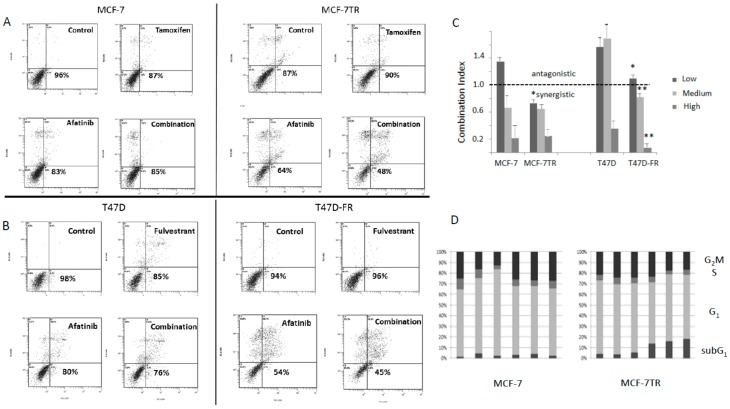
Flow cytometry data using annexin V (conjugated to fluoroscein isothiocyanate—FITC) (FL1) and propidium iodide (PI) (FL3) showing the effect of afatinib in combination with anti-oestrogens (48 h treatment) in MCF-7 parent and MCF-7-TR (tamoxifen-resistant) cell lines (**A**), and in T47D parent and T47D-FR (fulvestrant-resistant) cell lines (**B**) using the lowest dose levels of drugs (2.5 µM afatinib, 5.0 µM tamoxifen, or 0.5 µM fulvestrant). The lower left-hand quadrant of each representative data point cytogram represents the live cell population (FITC- and PI-negative). The percentages displayed indicate the live cell component in the given examples; (**C**) shows the combination index (CI) values obtained for the various drug treatments at three different levels (afatinib: 2.5 µM, 5.0 µM, and 10.0 µM; tamoxifen: 5 µM, 10.0 µM, and 20.0 µM; fulvestrant: 0.5 µM, 1.0 µM, and 2.0 µM). Comparisons between parent and resistant cell line CI values were made by two-way ANOVA with Bonferroni correction: * *p* < 0.001; ** *p* < 0.0001. (**D**) The various phases of the cell cycle according to drug treatment; data shown are representative of repeat experiments (*n* = 4). See Section 2.8 for statistical analysis.

Parental and anti-endocrine-resistant cells were treated with afatinib, tamoxifen, or fulvestrant singly or in combination (Figure 7). Using isobologram analysis, the combination index (CI) was calculated using the viability data (non-apoptotic population) obtained from the bottom left-hand quadrant of the cytograms obtained for the annexin assay shown in Figure 7A,B. In Figure 7C, the dotted line indicates CI values either as antagonistic (i.e., >1.0) or synergistic (<1.0). Looking at the panels of Figure 7A,B; it can be seen that for parental cell lines at the dose used (low dose), the effects were antagonistic (see Figure 7C). Synergistic effects were seen for both the parental cell lines at higher doses. However, there was clear evidence for greater synergistic effects of anti-oestrogens and afatinib in the drug-resistant versus parental cell lines, most significantly in the T47D-FR cells. For MCF-7-TR, the CI values were similar to those found for parental MCF-7 cells, but synergism was seen at all dose levels for the former. These data are indicative of the drug combination overcoming anti-endocrine resistance, particularly for the T47D-FR line.

### 2.8. Treatment of Anti-Endocrine-Resistant Cell Lines with Anti-Oestrogens and TKIs Produced Increases in the Sub-G1 Component of the Cell Cycle

Treatment of MCF-7 parental cells with either tamoxifen or fulvestrant gave rise to increases in the G1 component of the cycle, an effect not seen for the drug-resistant variant MCF-7-TR (with cross-resistance to both agents). Higher levels of cells in the sub-G1 fraction of the cell cycle were apparent for MCF-7-TR when treated with afatinib and either combination of tamoxifen or fulvestrant with afatinib (Figure 7D). Statistical analyses of the cell-cycle differences for MCF-7 versus MCF-7-TR were * *p* ≤ 0.05 for the G1 phase (tamoxifen and fulvestrant treatments) and ** *p* ≤ 0.01 for the sub-G1 fraction (afatinib and combination treatments) using a paired *t*-test with Bonferroni correction.

## 3. Discussion

Compelling clinical evidence suggests the HER tyrosine kinase receptor family plays a role in mediating anti-endocrine resistance [16]. Increased expression of growth factor receptors, particularly HER1/HER2, has been associated with both experimental and clinical endocrine therapy resistance [17]. Consequently, targeting these pathways using TKIs is a valid approach to reduce anti-endocrine-resistant cancer cell survival. It has been postulated that up-regulation of HER growth factor receptor signalling pathways leads to the modulation of ERα activity by causing ligand-independent receptor activation, resulting in enhanced ERα transcriptional activity [18]. Data provided by the current study give evidence for crosstalk between these two receptor signalling pathways, supported by others [19].

In the clinical setting, HER protein expression was associated with tamoxifen resistance in breast tumours from 205 patients with metastatic disease [5]. A preclinical study supported our finding that targeting EGFR signalling using the TKI gefitinib can decrease tamoxifen- and fulvestrant-resistant cell growth, and further it has been shown to delay acquired resistance in MCF-7 cells [20]. Cui et al. [21] studied HER2-mediated tamoxifen resistance and suggested a potential role for TKIs in blocking HER2 pathways to manage this type of endocrine-resistant breast cancer. A randomized phase II trial showed that afatinib compared favourably with lapatinib [22], and, hence, both agents were explored in the present study. HER3 lacks tyrosine kinase activity and is considered a defective protein [23]; however, its ability to form a heterodimer with HER2 could still implicate it in the resistance mechanism. In line with our findings for MCF-7-TR (tamoxifen-resistant) cells, others have reported that tamoxifen treatment can induce HER3, which is then implicated with tumours more prone to relapse [24].

Recent reports indicate that whilst fulvestrant effectively inhibits ERα-dependent cell growth, which may be low in the presence of drug resistance and cell survival, it also gives rise to enhanced HER signalling and ligand expression [25,26].

Only one previous study explored the relationship between ERα status and lapatinib treatment to reverse endocrine resistance when using tamoxifen-resistant MCF-7 cells with long-term oestrogen depletion [27]. The same study also showed that lapatinib reactivated ERα genomic function, observed by increased ERα transcriptional activity in tamoxifen-resistant models [27], in support of the present study. Various data obtained in our study indicated a profound effect on the reduction of cell viability with reactivation of ERα at the genomic level in fulvestrant-resistant cells when treated with the combination of TKI and anti-oestrogen, with clear evidence of synergistic drug effects.

An important question regards the mechanism underlying the sensitivity of endocrine-resistant cells to TKIs. To answer this question, we measured the expression of CDKI p27^kip1^. Significant induction of p27^kip1^ was observed when both resistant cell lines were treated with lapatinib. In contrast, p27^kip1^ expression was abolished when these cells underwent treatment with tamoxifen. Loss of p27^kip1^ leads to abrogation of cell-cycle arrest and mediates cell progression and drug resistance. These findings might explain why lapatinib caused cell death, as a massive induction in p27^kip1^ is required to oppose p27^kip1^ proteolysis and cause cell-cycle arrest in tamoxifen-resistant cells [28]. To date, our finding of the induction of p27^kip1^ occurring in fulvestrant-resistant cells following treatment with lapatinib is novel. Skp2 is required for the ubiquitination and consequent degradation of p27^kip1^, which allows cells to enter the S-phase. This study observed non-detectable levels of Skp2 in the parental MCF-7 and T47D cells (in the absence or presence of anti-oestrogens). In contrast, the expression of Skp2 was markedly increased in tamoxifen- and fulvestrant-resistant cells, offering an explanation for their low expression of p27^kip1^. A previous study has also shown that low p27^kip1^ and high Skp2 are more frequently associated with poor prognosis among breast cancer patients [29,30]. In addition, it has been reported that overexpression of Skp2 is associated with resistance to doxorubicin-based chemotherapy in breast cancer [31], which further suggests its potential as a therapeutic target [30]. Other signalling pathways may also have played a role for the effects observed in our study; for example, both MCF-7-TR and T47D-FR showed significantly upregulated activated PI3-kinase (data not shown; manuscript in preparation).

The cell-cycle effects we saw in MCF-7-TR tamoxifen-resistant cells were in keeping with a report by Suzawa et al. [32], who showed induction of apoptosis in lung cancer cells with HER2 mutations with exquisite sensitivity to afatinib. In that study, the cell lines showed G1 arrest and sub-G1 arrest following drug treatment. It should be noted that the cell line used in that study was exquisitely sensitive to the effects of afatinib because of a mutation in HER2, which was different from the cell lines used in the present study.

In conclusion, anti-endocrine resistance is associated with upregulation of the EGFR/HER2 signalling pathway. The crosstalk between these pathways as a mechanism for the development of endocrine resistance provides a rationale for using combinations of therapies.

Interrupting this crosstalk with TKIs can inhibit the survival signalling of EGFR and HER2 and enhance cell-cycle arrest via induction of P27^kip1^, leading to catastrophic apoptotic cell death.

## 4. Materials and Methods

### 4.1. Chemicals and Reagents

All reagents for Western immunoblotting used the NOVEX NuPAGE gel system with Western Breeze chemiluminescent immunodetection secondary antibodies (Invitrogen, Paisley, UK). Primary antibodies for HER1, #2232 and #2235; HER2, #2165 and #2247; HER3, #12708 and #2842; and ERα, rabbit monoclonal antibodies #8644 and #2517, were from Cell Signalling Technology. The antibody to P27^kip1^ (sc-1641) was from Santa Cruz Biotechnology, and TRIzol for RNA extraction was purchased from Life Technologies. 4-Hydroxytamoxifen, obtained from Sigma Aldrich, was dissolved in 100% ethanol and stored at −20 °C; 3-(4,5-dimethylthiazol-2-yl)-2,5-diphenyltetrazolium bromide (MTT) was purchased from Sigma Aldrich (St. Louis, MI, USA). Lapatinib, afatinib, and gefitinib were manufactured by Selleck Chemicals (Houston, TX, USA) and dissolved in DMSO as stock solutions; the SYBR Green-based real-time qPCR (RT-qPCR) assay was used for gene expression analysis primers: human oestrogen receptor 1 (*ESR1*) (NM_000125; product PPH05884A) and *GAPDH* (Qiagen, Hilden, Germany).

### 4.2. Cell Culture

The hormone-responsive cells MCF-7 and MDA-MB-231 triple-negative breast cancer cells (HER1 over-expressing positive control) were obtained from ATCC (LGC Promochem, Teddington, UK), and T47D and SKBR3 (HER2 over-expressing positive control) were obtained from the European Collection of Cell Cultures (ECACC) (Porton Down, Salisbury, UK). The novel drug-resistant cell lines for MCF-7-TR and T47D-FR were developed by culturing in increasing doses of the appropriate selecting drug until a stable resistance phenotype was acquired. The development of drug-resistant MCF-7-TR cells was carried out according to the protocol described by Samadder et al. [33], using a maintenance dose of 5 µM 4-hydroxy-tamoxifen, equating with a level of tamoxifen that is clinically achievable in breast cancer tissues [34]. For T47D-FR, the maintenance dose of fulvestrant was 0.4 µM. All cell lines, with the exception of ER-positive cell lines, were cultured in Dulbecco’s modified Eagle’s medium (Sigma Aldrich, St. Louis, MI, USA) with 2 mM Glutamax and supplemented with 10% heat-inactivated foetal bovine serum (FBS) (Invitrogen, Paisley, UK) at 5% CO_2_/37 °C. The ER-positive cell lines MCF-7 and T47D were cultured in phenol-red free DMEM/F12 medium (Sigma Aldrich, St. Louis, MI, USA) containing 10% dextran-coated charcoal stripped FBS (Invitrogen, Paisley, UK). MCF-7 and T47D cells were routinely treated with oestradiol (E2) (1 nM) (Sigma-Aldrich, Poole, UK) after each passage. All cell lines were monitored for the presence of mycoplasma during the study.

### 4.3. Cytotoxicity Testing

Using a MTT assay, cultured cells in single-cell suspensions were seeded into a 96-well plate at a density of 0.5 × 10^4^ (MCF-7 and MCF-7-TR) or 0.8 × 10^4^ (T47D and T47D-FR) cells per well. Cells were allowed to grow and attach in a humidified incubator for 24 h. Non-drug-treated wells were used as controls. Cells were incubated with the drugs, and DMSO solvent was added for control (untreated) wells for 72 h (MCF-7) or 96 h (T47D). To evaluate cell viability, 20 μL of MTT dye (5 mg/mL in PBS) was added to each well. After 4–6 h incubation, the medium was aspirated, and formazan crystals were dissolved in 200 µL of DMSO; the absorbance was detected at 540 nm using a plate-reading spectrophotometer. Dose-response curves were created in GraphPad, and IC_50_ values were calculated by fitting with a four-parameter model.

### 4.4. Western Blot Analysis 

Whole-cell lysates were made from monolayers of cultured cells in the exponential phase of growth. Cells were subjected to various treatment protocols (as indicated in the figure legends) after seeding into tissue culture flasks at 30–40% confluence. Cells were harvested at appropriate time points using a non-enzymatic cell dissociation fluid, washed twice in PBS and resuspended in RIPA buffer. Whole-cell lysate protein was loaded onto SDS-PAGE gels, electrophoresed, and Western transferred to a PVDF membrane. Probing of the membranes for GAPDH levels to act as loading controls was carried out by membrane stripping with 1 mM sodium azide in PBS for 1 h, followed by incubation using a murine antibody (Sigma Aldrich, St. Louis, MI, USA). Visualisation was carried out using alkaline phosphatase-conjugated secondary antibody (WesternBreeze^®^, Life technologies, Paisley, UK) with chemiluminescent substrate (CDP Star^®^, Invitrogen, Carlsbad, CA, USA). Protein expression levels were quantified by use of densitometry using chemiluminescent film, and processing of the scanned images as JPEG files was performed using Quantiscan 3.0 software (Biosoft^®^). Band intensity was normalised to GAPDH and was expressed relative to MCF-7/T47D controls (values shown are the means ± SD of three independent experiments, with *p*-values shown in the legends).

### 4.5. Real-Time Quantitative PCR Assay

RNA extraction was carried out using TRIZOL (Life Technologies, Carlsbad, CA, USA) followed by cDNA synthesis using the ImProm II reverse transcription system (Promega, Madison, WI, USA). RT-qPCR was performed using QuantiTect^®^ reagent (Qiagen, Hilden, Germany) and an ABI Prism 7700 Sequence Detection System (Applied Biosystems, Foster City, CA, USA). Reactions were prepared in 96-well plates in duplicate; using RT^2^-qPCR primers for the *ESR1* gene of interest (GOI) and GAPDH for the housekeeping gene (HKG). The programme applied as recommended by the manufacturer was as follows: 10 min at 95 °C PCR for initial activation, 40 cycles for denaturation for 15 s at 95 °C, and annealing for 60 s at 60 °C. The fold change in gene expression was determined by using the formula 2^(−∆∆Ct)^, where ∆∆C_t_ is the ∆C_t(gene of interest)_ − ∆C_t(GAPDH)_ and C_t_ is the threshold cycle at which the fluorescence passes the threshold.

### 4.6. Optical and Fluorescence Confocal Microscopy (Confocol Laser Scanning Microscopy)

All fluorescent dyes were obtained from Molecular Probes (Invitrogen, Carlsbad, CA, USA). For a typical experiment, cells were grown on a cover slip in a 6-well plate at a density of 3 × 10^5^ cells per well for the parent and 4 × 10^5^ cells per well for the resistant sub-lines. Cells were incubated overnight at 37 °C to allow for attachment and treated with drugs (10 µM tamoxifen, 1 µM fulvestrant, and 5 µM lapatinib) for 48 h. Cells were then covered with methanol for 20 min to fix, further washed with PBS, and incubated with 0.1% Triton X-100 to permeabilize cell membranes. Slides were incubated in 0.1% BSA in PBS/Triton for 1 h to block non-specific binding, followed by overnight incubation with the respective primary antibody (HER1 or HER2, as used for Western blotting) at a dilution of 1:200 in PBS/Triton at 4 °C. Cover slips were then washed with PBS and incubated with the secondary antibody conjugated to Alexa-Fluor488 for 2 h. Following PBS rinsing, the nuclear dye TOPRO3 was applied for 10–20 min. Slides were air-dried, and coverslips were added using the VECTASHIELD mounting medium. The edges of the slides were sealed with commercially available nail lacquer. Confocal microscopy was performed using a Zeiss LSM 510 confocal microscope. TO-PRO-3 nuclear stain was excited at 633 nm using an Argon/2 laser, and emission was detected between 650 and 710 nm. Alexa Fluor was excited at 488 nm and detected at 500–550 nm. The settings for the instrument were kept constant for all experiments.

### 4.7. Annexin V with Propidium Iodide Methodology for Apoptosis with Flow Cytometry

Cells were seeded into tissue culture flasks to give approximately 50% confluence, allowed to attach for 2–3 h, and then treated for a period of 48 h with either tamoxifen or fulvestrant in combination with afatinib. Each drug was administered at three dose levels, giving a fixed ratio (i.e., afatinib: 2.5 µM, 5.0 µM, and 10 µM; tamoxifen: 5 µM, 10 µM, and 20 µM; fulvestrant: 0.5 µM, 1.0 µM, and 2.0 µM). An annexin V–fluoroscein isothiocyanate (FITC)-conjugated apoptosis detection kit incorporating propidium iodide (PI) was used as described by the manufacturer’s protocols (Merck, Middlesex, UK). Harvesting of the cells included collection of attached cells following trypsinization as well as floating cells, which were combined in the sample for processing. Analysis by flow cytometry used the FL1 (FITC) and FL3 (PI) laser lines, and each sample was assessed using a collection of 10,000 events. For isobologram analysis, the Calcusyn program (version 211) was used (Biosoft^®^). Analyses were set up on three separate occasions.

### 4.8. Cell-Cycle Analysis (Flow Cytometry)

Cells were treated in flasks and harvested at 48 h by trypsinization and pelleting in PBS. Cells were then mixed by vortex with the drop-wise addition of 70% cold ethanol for fixation. Suspensions were stored for at least 24 h and were then washed twice in PBS and treated with ribonuclease A (Sigma, St. Louis, MI, USA) at 500 μg/mL for 30 min at 37 °C. PI solution was added from 50 μg/mL stock and left for 30 min prior to analysis on a Coulter EPICS flow cytometer using the L3 laser line. Cell-cycle phases were obtained using the MultiCycle software.

## 5. Conclusions

With a high proportion of breastcancer patients being maintained on anti-endocrine agents such as tamoxifen and fulvestrant, the issue of the long-term use of these agents and the development of drug resistance is of importance. Strategies to circumvent drug resistance are necessary, and the data described in the present paper suggests a strategy incorporating anti-endocrine agents and HER-directed therapies. We show evidence for synergy between the HER-directed agents afatinib and tamoxifen as well as fulvestrant in breast cancer cells with acquired anti-endocrine resistance. The data obtained are supportive of clinical trials that address the problem of acquired anti-endocrine-resistant breast cancer using combinations of these agents.
